# Policy context and narrative leading to the commissioning of the Australian Indigenous Burden of Disease study

**DOI:** 10.1186/s12961-015-0004-0

**Published:** 2015-03-15

**Authors:** Jessica R Botfield, Anthony B Zwi, Peter S Hill

**Affiliations:** Health, Rights and Development, School of Social Sciences, The University of New South Wales, Room G25, Morven Brown building, UNSW, Kensington, 2052 NSW Australia; School of Public Health, University of Queensland, Room 118, Public Health Building, Herston Rd, Herston QLD, 4006 Brisbane, Australia

**Keywords:** Australia, Burden of Disease, Health policy, Indigenous disadvantage, Research translation

## Abstract

**Background:**

Burden of disease (BoD) studies have been conducted in numerous international settings since the early 1990’s. Two national BoD studies have been undertaken in Australia, in 1998 and 2003, although neither study estimated the BoD specifically for Indigenous Australians. In 2005 the Australian Government Department of Health and Ageing Office for Aboriginal and Torres Strait Islander Health formally commissioned the University of Queensland to undertake, in parallel with the second national BoD study, the “Burden of Disease and Injury in Aboriginal and Torres Strait Islander Peoples” study, drawing on available data up to 2003. This paper aims to explore the policy context and narrative in the lead up to commissioning the Indigenous BoD (IBoD) study, focusing on relevant contextual factors and insights regarding the perspectives of key stakeholders and their anticipated value of the study. It is part of a broader project that examines the uptake of evidence to policy, using the IBoD study as a case study.

**Methods:**

A systematic review of the literature was undertaken in late 2013 and early 2014, and the findings triangulated with 38 key informant interviews with Indigenous and non-Indigenous academics, researchers, statisticians, policy advisors, and policymakers, conducted between 2011 and 2013.

**Findings:**

Contextual features which led to commissioning the IBoD study included widespread recognition of longstanding Indigenous disadvantage, lower life expectancy than non-Indigenous Australians, and the lack of an adequate evidence base upon which to determine priorities for interventions. Several anticipated benefits and expectations of key stakeholders were identified. Most informants held at least one of the following expectations of the study: that it would inform the evidence base, contribute to priority setting, and/or inform policy. There were differing or entirely contrasting views to this however, with some sharing concerns about the study being undertaken at all.

**Conclusions:**

The IBoD study, in concept, offered the potential to generate much desired ‘answers’, in the form of a quantified ranking of health risks and disease burden, and it was hoped by many that the results of the study would feed into determining priorities and informing Indigenous health policy.

## Background

This paper describes and analyses the commissioning of the Australian Indigenous Burden of Disease (IBoD) study published in 2007. We examine the lead-up to its commissioning and the expectations of the range of stakeholders involved. Before presenting this case study, we offer a brief overview of the Global Burden of Disease (GBD) project.

The first GBD study, initiated in 1992 at the request of the World Bank [1,2], was a major milestone in global health and development, and an integral component of the 1993 World Development Report [3]. The GBD and its derivatives have undoubtedly influenced conceptualisation of the global health agenda, the determination of health and related research priorities, and the allocation of resources [4,2,5]. The underpinning concepts, metrics, and their implications have continued to be critiqued, debated, and further developed. The most recent GBD study was published in 2015, drawing on 2013 data [6].

Burden of Disease (BoD) seeks to quantify the loss of healthy life from disease and ill-health, and is conventionally measured in disability-adjusted life years (DALY) [3] and derivatives of these measures. The DALY is a time-based measure that combines years of life lost due to premature mortality and years lived with a disability, metrics specifically developed to assess the BoD [7]. The DALY was first developed in 1992 and has since been widely applied to measure the BoD in populations [8]. The total loss of DALYs globally is referred to as the GBD [3], and findings are typically presented in a variety of ways including by region, country, and risk factor.

The GBD methodology and assessment of DALYs did not go unchallenged, when first developed, with critics identifying a number of problems, both conceptually and empirically. While some of these concerns have been addressed in subsequent versions of the GBD and its measurement and presentation, others remain. Early criticism of the GBD and BoD approaches suggested the conceptual and technical basis for DALYs was flawed, its assumptions and value judgements open to question [9], and that the proponents of DALYs did not distinguish between measuring the BoD and allocating resources [10]. Others argued that not all disease burden could be estimated, and that the investment needed for BoD studies would divert resources from other work and focus attention on major diseases, with limited linkage to interventions [1,11]. Reidpath et al. [12] suggested that the lack of consideration of realistic contexts would result in a measure that underestimates the burden associated with morbidity in disadvantaged populations and overestimates the burden in advantaged populations. More recent critiques highlight the continued uncertainties around health data and the future ideal to properly count and account “*for all the world’s citizens, so that complex estimation techniques are not needed*” [5]. How data from under-serviced communities are accurately reflected remains an issue of concern.

Following a major revision of the GBD, a comprehensive re-estimation of disability weights was provided for the full set of around 230 unique sequelae associated with the array of disease and injury causes in the study [4]. The GBD Study 2010, released in late 2012, provided regional estimates of deaths and DALYs (using a new method for calculation of DALYs). It was at that stage the most comprehensive GBD study, producing comparative metrics for 291 different causes of premature death and disability across 187 countries for the years 1990, 2005, and 2010 [13]. Due to improved definitions, methods, and data, the results supersede all previously published GBD results. The Australian BoD studies – national, state and Indigenous – precede these modifications.

National BoD studies have been conducted in a range of countries. Murray et al. [14], in their analysis of the GBD Study 2010, cite 59 papers written on BoD studies conducted in Australia, South America, the United States, Europe, Asia, and Africa. Such studies reveal a degree of applicability to national health policy and planning activities [15-17]. However, it is notable that, whilst BoD studies have been conducted on a global and national level for the past two decades, fewer sub-national studies have been undertaken and the IBoD study appears to be the first BoD study conducted with a specific focus on an Indigenous or minority population.

## Australian burden of disease studies

A national BoD and Injury study was first conducted in Australia in June 1998, using the GBD methods adapted to the Australian context and drawing on Australian sources of population health data from 1996 [18,19]. A Victorian state-based BoD study was also undertaken in 1998, to provide a comprehensive assessment of premature mortality, morbidity, and disability attributable to diseases, injuries, and various risk factors in 1996, with projections to 2016 [20]. This study was then updated for the year 2001, to provide a second comprehensive assessment of the health status of the Victorian population [21].

The BoD framework was not specifically applied to the Australian Indigenous population in the national study, nor was it a focus of subsequent state-based studies [22]. These earlier studies did not quantify the BoD for Indigenous Australians due to concerns about incomplete Indigenous identification data in population datasets [22]. It was a pilot BoD study in the Northern Territory, published in 2004, that first focussed on both Indigenous and non-Indigenous people [23]. This study used data from 1994 to 1998 and argued that it was able to largely overcome data constraints – it could access Indigenous identification in both death certification and most sources of information on morbidity. It provided comparative data to identify health priorities and facilitate more effective and efficient resource allocation, although the study remained ‘exploratory’ due to concerns about potential inaccuracies in the available data, with the quality of data sources ranging “*from ‘excellent’ in disease surveillance systems to ‘reasonable’ for the extrapolation method of national averages*” [23].

A second national Australian BoD study commenced in 2003 to update and expand on the first [17]. It was during this time that the Australian Government Department of Health and Ageing (DOHA) Office for Aboriginal and Torres Strait Islander Health (OATSIH) began negotiations with the University of Queensland (UQ) to undertake, in parallel to the second national study, an estimate of the BoD in Indigenous Australians, also drawing on available data up to 2003 [22]. This was formally commissioned in 2005, and took advantage of the well-established and internationally recognised team working at the UQ at the time. While the Indigenous component was investigated at the same time as the national BoD study, findings and analyses were published separately in a companion report as the ‘Burden of Disease and Injury in Aboriginal and Torres Strait Islander Peoples 2003’ (hereafter referred to as the ‘Indigenous Burden of Disease (IBoD) study’). The work was finalised in 2007 and published in a 2007 report [22] and a peer-reviewed paper in 2009 [8].

## Methods

The present paper reports on one component of a larger NHMRC-funded study on evidence and policy in Aboriginal health, using the IBoD study as a case study for analysis. The objectives of the broader study were to i) explore how meaning is constructed from the IBoD research evidence by different policy stakeholders; ii) consider prioritization through BoD with other approaches in priority setting; iii) map out the implications for the use of different data, frameworks, and approaches for priority determination and agenda setting; iv) identify ways to enhance the use of research evidence in policy; and v) extend the current theoretical base of health policy analysis.

This particular paper draws on data collected from a literature review, a systematic review, and key informant interviews, to elucidate the story behind the IBoD study – why it was commissioned, the context in which this occurred, and the expectations of the different stakeholders involved. It complements our study on the measurement and presentation of the life expectancy gap in Indigenous health [24] and will sit alongside related papers examining the process and findings of the IBoD study.

A background literature search for relevant ‘grey literature’ was undertaken in 2013. Key materials from the IBoD study itself were also accessed, including, for example, the Agreement between DOHA and UQ, and agendas and minutes from Steering Committee (SC) and Technical Advisory Panel (TAP) meetings. In our Discussion, we refer to literature and frameworks from Bowen and Zwi [25], Head [26,27], Walt [28], Nutbeam and Boxall [29], and Kingdon [30] to reflect on the role of research and evidence in the policy process.

The systematic review undertaken in November 2013 utilised Scopus, Informit e-library, and ProQuest Central to identify all literature that referenced the IBoD study in Australia, to assess how and in what ways data has been used and/or taken up in policy and/or practice. The search strategy utilised a combination of the following search terms: ‘Burden of disease and injury in Aboriginal and Torres Strait Islander Peoples’; [OR] ‘Burden of disease’ AND ‘Aborigin*’. No inception date was chosen to ensure all relevant publications were captured; all materials up until the search on 5 November 2013 were included. Grey literature was also included from searches within key institutions and organisations^a^ from 2007 to date. Findings from this systematic review are being written up in a separate paper; here, we draw on a small number of the relevant papers identified.

A total of 38 key informant interviews were conducted between 2011 and 2013 with Indigenous and non-Indigenous academics, researchers, statisticians, policy advisors, and policymakers. The interviews focussed primarily on the background to commissioning the IBoD study and expectations regarding its relationship to policy and practice. The subjects for interview were identified using ‘events-based sampling’ [31]; the ‘event’ being the commissioning and implementation of the IBoD study, and the critique on its release. The sample comprised members of the IBoD research team, SC and TAP members, overt critics of the research, and those responsible for applying its findings into policy. All identifiable stakeholders directly involved in the process were considered relevant informants, and were contacted by email or telephone and invited to participate. The response rate was high, with only two declining and one stakeholder deceased.

Interviews were semi-structured, using question lines developed by team members, and were conducted face to face, and in a small number of cases, by telephone. Consent for the interview was noted on paper or at the commencement of the interview, which was recorded and subsequently transcribed. Informants were divided into five groups for the analysis (academic, member of IBoD research team, statistician, policy advisor, or policymaker), and NVivo 10 [32] was used for organising and coding the data. Coding involved independent reading of all interview transcripts by two members of the research team, who developed a coding frame based on the major themes emerging. Coding of all interviews was undertaken by one person in NVivo 10 [32], who identified additional codes as needed. Upon completion of this, the coding was independently checked by another person, and consensus on final themes and associated codes was agreed.

Ethics approval was obtained from the Behavioural and Social Sciences Ethical Review Committee at the UQ (approval number 2010001442).

This paper is structured around answering the following questions:

i. What were the main contextual factors that led to the commissioning of the IBoD study?
ii. How was the IBoD study commissioned, and who was involved in this process?
iii. What were the expectations of stakeholders in relation to the IBoD study, in the lead up to and during the commissioning?
iv. How does this case study relate to the literature on the use of evidence in the policy process?

This paper critically examines the background and policy context, preliminary negotiations, and the narrative culminating in the commissioning of the IBoD study. We consider the motivation behind the study and the anticipated value of the study by key stakeholders. The paper focuses primarily on three key areas: the contextual factors in the lead up to the commissioning of the IBoD study, the commissioning process itself, and the expectations of stakeholders.

## Findings

### Contextual factors leading to the commissioning of the Indigenous Burden of Disease (IBoD) study

#### Indigenous health in the 1990’s and early 2000’s

The context in which the IBoD study was commissioned was characterised by widespread recognition of Indigenous disadvantage and inequities in health and its determinants. High levels of inequalities in mortality, morbidity, life expectancy, and a raft of other health indices had been well documented [33-35]. The infant mortality rate for the Indigenous population was almost three times that of the general Australian population [35,36]. While precise life expectancy rates and the gap between Indigenous and non-Indigenous Australians are contested [24,37], in 2005 the gap was estimated to be “*about 17 years*” [38]. The Australian Bureau of Statistics (ABS) released their inaugural biennial report ‘The Health and Welfare of Australia’s Indigenous Peoples 2003’ in 2003, which argued that Indigenous Australians had a life expectancy that “*is about 20 years shorter*” than non-Indigenous Australians, and suggested that Indigenous people suffer disproportionately from the consequences of European settlement, with many living in conditions of economic disadvantage due largely to lower education and employment levels [36]. As commented by McMurray [37], the 17-year difference in life expectancy, based on the 1996–2001 estimates, became well known and was important in the development of Indigenous policy by Australian governments.

National concern regarding the inequitable state of Indigenous health in the early 2000’s was apparent, with both government and the public health community concerned. One academic interviewed noted that: “*Australia was a kind of international pariah, for its failure to tackle Aboriginal health. We had gone through decades of the era of what I call unfunded policy. Aboriginal Health is bad*” and “*Aboriginal health in this country has been, arguably, the biggest public health failure in the western world*”. Political pressure was clearly mounting, with numerous organisations and individuals seeking improvements in Indigenous health: “[Australia] *would pontificate all around the world about human rights, and other people would say… if you were really serious about human rights you would sort out the mess in your own backyard*” (Academic). A policy advisor stated that: “*…for a long time* [there has] *been a strong will on the part of governments of either political persuasion to improve Aboriginal and Torres Strait Islander health, and a desire on the part of the Australian Department of Health and Ageing to be improving Aboriginal health*”.

Table 1 provides additional context and highlights this ongoing interest and concern around Indigenous health through a chronology of key policies, reports, and events from 1998 to 2013.

**Table 1:** **Chronology of relevant policies, reports, and events (1998–2013)**

**Year**	**Australian BoD studies**	**IBoD study**	**Related policies & reports**	**Relevant events**
1998	First Australian & Victorian BoD study undertaken			
1999	First Australian & Victorian BoD study published			
2000			Final report of the Council for Aboriginal Reconciliation to the Prime Minister and the Commonwealth Parliament delivered (December)	
2001			*National Health Performance Framework* released (September)	Federal election (November)
2002				COAG agreed to commission the SCRCSSP to produce a regular report to COAG against key indicators of Indigenous disadvantage, to measure impact of policy changes on Indigenous community (April)
2003	Second Australian BoD study undertaken		Inaugural biennial report *The health and welfare of Australia's Indigenous Peoples 2003* published (ABS & AIHW)	
			*Overcoming Indigenous disadvantage: key indicators 2003* report published (SCRCSSP)	
2004	First Northern Territory BoD study published	Commissioning of IBoD study		Federal election (October)
				Governmental changes regarding a ‘whole-of-government’ approach to Indigenous affairs at Federal level implemented (July)
				The *2002 National Aboriginal and Torres Strait Islander Social Survey* released (ABS, June)
2005	Second Victorian BoD study published		*Health and welfare of Australia's Aboriginal and Torres Strait Islander peoples, 2005* published (ABS & AIHW, August)	
			*Social Justice Report 2005* published	
2006				
2007	Second Australian BoD study published	IBoD study report published	*Little Children are Sacred* report published (June)	Federal election (November)
	Second Northern Territory BoD study undertaken		Oxfam *Close the gap: solutions to the Indigenous health crisis facing Australia* report released (April)	Northern Territory Intervention (June 07–October 08)
				Australian DOHA: *National Strategic Framework for Aboriginal and Torres Strait Islander Health 2003–2013: Australian Government Implementation Plan 2007–2013*
2008			*National Indigenous Reform Agreement* (November)	National Apology (February)
			*Indigenous Chronic Disease Package* (November)	Close the Gap: Indigenous Health Equality Summit – Statement of Intent (March)
			Closing the Gap framework (December)	National Partnership Agreement on Closing the Gap in Indigenous Health Outcomes (December)
2009	Second Northern Territory BoD study published	Peer-reviewed paper published (April)	*National Health Performance Framework* revised	New life expectancy estimates released (ABS, May)
2010			*Closing the Gap: The Indigenous Chronic Disease Package in 2009–10* (first annual report)	
2011			*2010 Indigenous Expenditure Report* (first edition) (February)	
			*Closing the Gap: The Indigenous Chronic Disease Package in 2010–11* (second annual report) (October)	
2012			*Aboriginal and Torres Strait Islander Health Performance Framework*	
			*2012 Indigenous Expenditure Report* (second edition) (September)	
2013			The *Aboriginal and Torres Strait Islander Peoples Recognition Bill 2012* passed (February)	Federal election (September); Liberal Coalition government elected under Prime Minister Tony Abbott
			*Closing the Gap: Prime Minister’s Report 2013* released	
			*Australian Aboriginal and Torres Strait Islander Health Survey: First Results, Australia, 2012–13* released (November)	

ABS, Australian Bureau of Statistics; AIHW, Australian Institute of Health and Welfare; BoD, Burden of disease; COAG, Council of Australian Governments; IBoD, Indigenous burden of disease; SCRCSSP, Steering Committee for the Review of Commonwealth/State Service Provision.

#### Lead-up to the commissioning of the study

Routine data collection systems often underestimated the true rates of ill health in Indigenous populations due to poor quality data, including inadequate Indigenous identification in health service utilisation records [22]. One policy advisor suggested that: “*the problem in Indigenous health was a lack of good knowledge of Indigenous health*” and another advisor that: “*… there was a recognition that our traditional measures were really not up to it. They just weren’t satisfactory*”. A policy advisor in the Northern Territory argued that: “*the issue of Indigenous identification has been a priority for the last 15 to 18 years, and some states really still can’t report*”. There had also been considerable uncertainty about the precise level of mortality, as well as disease incidence and prevalence, among Indigenous Australians. The need for good quality and accessible data on Indigenous peoples was evident – for benchmarking, to assess the effectiveness of programs and interventions, and to evaluate policies [36].

Given some uncertainties within Indigenous data, DOHA were keen to improve the evidence base, and an IBoD study provided an opportunity to do so and expand on knowledge of Indigenous health. The IBoD researchers argued that the “*Burden of disease estimates for Indigenous Australians…would help to identify those diseases and risk factors that are most responsible for the gap in health status between Indigenous Australians and the Australian population overall*” and that these data would add value: “*OATSIH recognised the value of funding a study that would improve the evidence base for determining the size and impact of health problems in the Indigenous population, using a ‘burden of disease’ methodology*” [22]. An Indigenous academic interviewed suggested that: “*from OATSIH’s point of view I think that they genuinely were interested in getting a better handle on the burden of illness in Aboriginal communities*”, and a policymaker that: “[OATSIH] *had made a very long term commitment… that we would work in a concerted way to try and improve the data holdings… Having that* [IBoD] *work done within Australia was part of our international obligations, but also would significantly improve what we were doing around health status…*”*.*

In 2003, it was considered opportune to garner insights into the state of Indigenous health by applying BoD metrics; this also took advantage of the presence of leading members of the GBD team being based at the UQ. According to one policy advisor “*…it was the logical step. Alan Lopez*^*b*^*does the global Burden of Disease study. He comes to Australia and does the Australian Burden of Disease study, and we were all sitting around, and we needed an Indigenous Burden of Disease study*”.

In addition, as noted previously, several national and state-based BoD studies had previously been conducted in Australia, with the first national report being: “*…very, very popular and… used hugely in policy, in ministerial briefings all over the place*” (Government statistician). The importance of these earlier experiences in promoting an IBoD study was also highlighted by other informants: “*Treasury were familiar already with the Burden of Disease concept and were quite taken with it*” (Policymaker) said one, while another commented that the earlier BoD study for the Victorian government was very well received: “*I think that was certainly looked at and…that was very, very well communicated I thought by the Victorian government and I think that was a key factor in selling the Burden of Disease work to the Commonwealth*” (Policy advisor).

### Commissioning of the IBoD study

#### Key stakeholders

Many individuals and organisations were involved in the commissioning and implementation of the IBoD study. OATSIH commissioned and funded the UQ to undertake the study, under the leadership of Professor Alan Lopez and Dr Theo Vos. The SC was formed in 2005 and comprised representatives from the National Aboriginal Community Controlled Health Organisation (NACCHO), OATSIH, the Australian Institute of Health and Welfare (AIHW), the ABS, UQ, University of Melbourne, University of Wollongong, Edith Cowan University, James Cook University, and the Northern Territory Department of Health and Community Services. A TAP was also formed in 2005, with a smaller team of members from the ABS, AIHW, Menzies School of Health Research, Queensland Department of Communities Office of Aboriginal and Torres Strait Islander Partnerships, and the Northern Territory Department of Health and Community Services. The SC and TAP are discussed in further detail later in this paper.

#### Existing relationships and networks leading to the commissioning

Indigenous and non-Indigenous academics and policymakers identified informal networks between the policy and research community as “*pretty powerful*”, “*pretty important in research policy exchange*”, and “*absolutely critical*”. Many of those interviewed noted that existing relationships, networks and advocacy all played a role in the commissioning of the IBoD study. One policymaker reinforced this: “*… who you know, who phones you up, who you meet at a conference, who gets your attention… it certainly helps*”*.*

One example of this is that Dr Vos, one of the lead authors of the IBoD study, had been involved in both the national and Victorian BoD study. Treasury were therefore familiar with him and the BoD concept, as noted by several informants. One policymaker suggested that it was “*probably a mixture of strong advocacy by the researchers and a view on the part of the* [DOHA employee] *that it would yield something useful… I think there was quite a sort of close relationship between the researchers and the* [DOHA employee]*. So there was probably a little bit of a sort of amicable agreement that this would be something worth doing*”. Professor Lopez, who provided oversight of the IBoD study and was Chair of the SC, was also known to senior employees of the DOHA, who were keen to undertake the second BoD national study in Australia.

#### Commissioning process

Commissioning the IBoD study in 2005 seems to have been a relatively simple addition to the national BoD activities already underway, and there was broad agreement by a range of informants that the Indigenous study was seen as a component of the national study – “*we didn’t even consider it a separate project; we considered it an element of what the overall study was going to do*” (Research team member); “*it really was a subset. We couldn’t have done the Indigenous study without first… doing the Australian one*” (Research team member); “*So once all the methodology for the first Australian study were updated into the second national study, then that provided the opportunity to do* [the] *Indigenous Burden of Disease study as a supplement to the second national study*” (Policy advisor); and “*The Indigenous study was funded by OATSIH and it was produced separately but within the envelope of the national Burden of Disease study*” (Government statistician).

One member of the IBoD research team confirmed that OATSIH contributed funds for the Indigenous component of the national BoD funding package, and noted that the research team agreed that whilst they could do the Indigenous component “*with difficulty*”, they “*would certainly see that as a priority part*”*.* This informant suggested the IBoD study “*wasn’t a purposeful ‘we will do a study of sub-populations’… But it was just that part of [*the*] funding package and interest came from OATSIH*”*.*

A series of five contracts were put together by DOHA, each for different components of research (including the national BoD study and the IBoD study), with different groups within DOHA having an interest in specific aspects of the research. This complex arrangement led to an Indigenous academic informant suggesting that the “*Government’s process of commissioning…* [the IBoD study] *was really poor*” and another informant that “*…the contracts were appallingly written*”. The incremental addition of the IBoD study may have contributed to the concerns of one government statistician that there was also inadequate consultation with the National Advisory Group on Aboriginal and Torres Strait Islander Health Information and Data: “*…there is no evidence here to me that that was done… I’m not surprised. Disappointed, but I’m not surprised*” (Government statistician).

#### Process to engage stakeholders and their planned involvement in the study

As previously mentioned, two groups were established to support the IBoD study: a SC to provide guidance and ensure the study provided relevant information for policymaking and advocacy, and a TAP to discuss methodological issues [22]. According to the contract between DOHA and the UQ, the “*Burden of Disease Steering Committee, appointed by the Participant (UQ), will be chaired by Professor Alan Lopez and consist of experts and representatives of national Indigenous community organisations as well as a representative from OATSIH, to be appointed by the Department. The SC will guide the study to ensure the study provides relevant information for policymaking and advocacy. The SC will also provide scientific guidance and scrutiny of the study team’s interpretation of the evidence. A technical subcommittee will be formed to discuss methodological issues*” (Unpublished data). This contract also stated that the UQ and AIHW must ensure that the study received guidance and intellectual input from Professor Lopez and Dr Vos, and must consult with OATSIH, both of which it did.

According to the contract, the UQ were also to consult with epidemiological experts and Indigenous community organisations at both national and state/local level. While epidemiological consultation did occur through the TAP and BoD networks, there is limited evidence of any formal engagement with Indigenous organisations, except perhaps through several SC members. The new Cooperative Research Centre for Aboriginal Health, in which the UQ was a partner, was to also provide expertise and foster collaboration with Indigenous community organisations (Unpublished data), which does not appear to have happened. A representative of NACCHO was nominated to the SC, and two senior Indigenous academics, active in policy, were also appointed.

Whilst consultation with Indigenous community organisations was written into the contract, there appears to have been relatively little Indigenous engagement during the commissioning process. Several informants subsequently aired concerns with this. An Indigenous academic stated *“I don’t know that the Indigenous issues were really front and centre in it”* and *“I think the fact that it was commissioned like it was… it was really, you know, the horse has bolted, and now we’ve got to find a way of getting some form of Indigenous engagement and endorsement in the process”.* This informant also suggested there was a “*lack of Indigenous leadership at the time of the project…*”.

Several informants also critiqued the IBoD research team for having inadequate insights into the specificities of Indigenous health research. A policy advisor interviewed suggested that the researchers “*… didn’t have a good understanding of the Indigenous research and political environment… [there was] a lack of political knowledge and links into the Indigenous research environment… And that engagement was then problematical, because they were seen as outsiders coming into a patch where people had 15, 20 years or more experience in research”*. An Indigenous academic also noted that “*the friability and the weakness of this network was there weren’t enough Indigenous players who were connecting to those other policy networks and saying, ‘Hang on, this is a bit of work that’s got some methodological problems, it’s got some data problems, but it is pointing to some very important stuff that we need to bring into the mix.’ And that’s the other thing you lose, if you don’t have good Aboriginal participation in the process. You lose all that network of connection*”.

According to the minutes of the first SC meeting in 2005, there was “*not an Indigenous person represented on the TAP. There was general agreement from the steering committee members that an Indigenous person should be invited to sit on the TAP so as to assist with the interpretation of technical issues.* [An SC member] *commented that an effort had been made to include an Indigenous person with epidemiological expertise on the TAP … but this had not been achieved due to the person’s time commitments*” [39]. One Indigenous academic was active in the TAP, but with policy, rather than epidemiological expertise. Whilst a key peak Indigenous body had been invited to sit on the TAP, they were not available to do so.

#### Opposition to the study

Several informants from at least one government agency indicated that concerns with the availability and quality of Indigenous data led them to be less involved in, or supportive of, the IBoD study. Many were uncertain whether existing data would support an IBoD study, given the poor quality of data, under-reporting (particularly in certain jurisdictions), and the inconsistent identification in other data sources. One government statistician stated *“I didn’t think that the data would support it”* and that *“the problem with Indigenous data is that it is beset by problems which really stem from changing rates of identification over time and it’s very hard to understand those things and to allow for the issues which vary from place to place and from time to time and from topic to topic”*. Minutes from the first meeting of the SC show one member suggested they were initially *“…sceptical of its applicability due to the wide-ranging and well-known deficiencies in Indigenous data”* [32]. Concerns were also expressed that the findings of the IBoD study may be unpredictable and disrupt accepted positions, with one academic apprehensive that *“this [IBoD] project, rather than helping, might screw… up [the current government estimates of the 17 year life expectancy gap]. That was our worry”*.

According to a policymaker interviewed, even while the commissioning process was underway, there continued to be some debate within OATSIH as to whether they should support the study or not: “*we, relatively early on, were involved in a process about whether or not we were going to invest in the Vos work, and there was quite a long debate about methodology… we’d already made a commitment by then, there was some pressure by some participants as to whether we should continue to support it, and we made a decision that we would*”.

## Expectations of stakeholders in the lead up to, and commissioning of, the study

Key informant interviews identified three broad but inter-related expectations of the IBoD study, with many stakeholders sharing at least one or more of these. These are closely related and included improving the evidence base, informing policy discussions, and contributing to priority setting.

A recurring theme across many interviews was around both the perceived lack of reliable data for Indigenous health, and the hope that the IBoD study would generate metrics to allow better interpretation and use of existing data. Informants noted that while improvement was needed, it was not only the generation of new metrics and analyses that was deemed important, but also enhancing its quality and reliability. A policy advisor and policymaker expressed these viewpoints: *“… it was a very clear recognition that we needed to improve our epidemiological analysis of the data we had, and what could be brought to bear on the range of data that was available*”; “*…we’d made a very long-term commitment… that we would work in a concerted way to try and improve the data holdings*”. An Indigenous academic also suggested that: “*from OATSIH’s point of view I think that they genuinely were interested in getting a better handle on the burden of illness in Aboriginal communities*”, and a policy advisor that: “*the Department are very wedded to improving the evidence base*”. Other informants articulated similar viewpoints, stating: “*we knew that … the better the evidence base, the better armed we will be*” (Policy advisor). It was argued by a policymaker that OATSIH had “*made a very long term commitment through some Commonwealth* [and] *state committees and through relationships that we had… that we would work in a concerted way to try and improve the data holdings … it was also quite a strong commitment around evidence-based policy and about being able to measure whether what you’re doing makes sense. And so always being able to search for how you can draw on data that’s real and meaningful and measurable was a real drive for us*”.

One policy advisor noted the value of quantification, both in relation to commissioning the work: “*So governments of all persuasions will invest in finding more about a problem through research into issues that have a particular quantitative bent*”, and in building on the particular strengths of the BoD methods: “*So I guess we were trying to quantify those things, which the methodology is particularly good at, and other forms of health data don’t – Indigenous health data don’t give us…*”. Numerical data were seen as being particularly powerful and potentially influential within the policy environment, by both a policymaker and an academic interviewed: “*…so always being able to search for how you can draw on data that’s real and meaningful and measurable was a real drive for us*” and “*I think the burden of illness… may be useful to all in advocacy in trying to raise awareness or something*”.

The value of quantifying and synthesizing a range of disparate data in order to shed light on differences and key areas of focus was also intimated by a member of the IBoD research team: *“Putting it all together, in a consistent framework where you can quantify it in the same way against each other and be able to say, these are the big health problems, at these ages, men versus women, remote versus non-remote, this disease versus that risk factor, now, that’s a huge step forward”*. Another informant emphasised the value of data that directly compared Indigenous and non-Indigenous experience, thus contributing to focusing on where change was needed: *“I think one of the valuable things that we were hoping to get out of it was just the Indigenous/rest of Australia comparison. So you could actually have some evidence as to where to focus to make a difference”* (policy advisor).

Informants identified the inherent attractions to a metric that helps generate a single number upon which estimates can be based and which can set priorities and inform policy. The value of numerical data was highlighted by one academic: “*You’ve got policymakers saying, ‘We’re not interested in your technical ifs and buts, give us the number’*”, implying that one could work with numbers but not uncertainty which often accompanies them. This reinforces a well-observed policy concern with quantification, as described in other Australian studies focused on other policy issues [40]. Another academic interviewed also noted concern about quantification, stating: “*I’m not opposed to quantification. Quantification can bring rigour. What I’m opposed to is quantifying for the sake of quantification or quantifying something over here because we can’t quantify* (t)*here*”.

Several informants did express concern about the quality of the data the IBoD study was to work with, and whether it would produce reliable results, as previously noted. One government statistician suggested “*the problem with the Indigenous Burden of Disease was really, the quality of the data was an issue. And it created quite a bit of angst among the Steering Committee in general*” and that “*the Indigenous issue is problematic because of the quality of the Indigenous data*”. Minutes from the first SC meeting showed that, while one member was initially sceptical of the applicability of the study due to “*the wide-ranging and well-known deficiencies in Indigenous data*” [39], they came to realise: “*…there was lots of value in going through the exercise and putting up estimates as one of the outcomes of the process would be identifying strengths and gaps in health datasets which would aid future studies in Indigenous health*” [39].

Two additional anticipated benefits that emerged from the interviews was the hope that the evidence from the IBoD study would inform policy and contribute to priority setting, as the study was: “*… policy relevant from the outset*” (Indigenous academic). The IBoD researchers themselves were confident of the pertinence of the research to policy and priority setting: “*I guess I hoped that it would be used as evidence in informing policies*”, and “*People working on it, I think, were passionate about these results* [and that they] *would influence policy, or these results would make a difference… it would give an overview, as I said, so then people could decide where to target resources to make the biggest difference*”*.* One senior policymaker also confirmed early expectations of its value: “[Treasury] *saw it as, even then, potentially useful for policy*”.

An opposing view about the study’s potential contribution to policy was articulated, however, with one academic suggesting the “…*development of policy is multi-faceted. The numerical data… they’re only a small part of the picture; it’s the political context and cultural context and economic setting, and all the rest of it, that is really fundamentally determinative. Put it this way, we don’t lack effective policies for Indigenous health in Australia because we lack data*”. Nevertheless, alongside this strongly held view, were repeated calls by other informants for more and better data.

For some academics and the IBoD researchers, the ultimate value of the IBoD study lay in its capacity to inform resource allocation: “*It’s about establishing and confirming priorities*”; “*it was a good and useful kind of thing in sort of priority setting and this sort of stuff*”; and “*then people could decide where to target resources to make the biggest difference*”. One policy advisor also suggested that the study might improve understanding of more neglected areas, such as mental health: “*… that’s where the Department, I’m sure, was seeking to draw on those areas where we didn’t have a good understanding. And the Burden of Disease methodology…* [would] *close the knowledge gap around mental health issues and so forth*”.

Not all informants held these views about resource allocation though, with one critic commenting that the IBoD study: “*…as a methodology for priority setting… it’s a waste of time and potentially misleading, because to know the size of a problem tells you nothing about what to do about it*” and that “*I think we knew* [the priorities] *before*” (Academic). Another academic similarly suggested that the focus should not be on establishing priorities to direct resources, but in identifying strategies that were going to make effective use of the resources, and that “*energy needs to be directed to the search for new and novel ways of addressing the problems*”.

## Discussion

Indigenous Australians suffer disproportionately to non-Indigenous Australians, often living in conditions of social and economic disadvantage, and experiencing worse health [38]. Despite acknowledgement of vast differentials in health status, evidence has been limited to traditional population health indicators. At the time the IBoD study was commissioned, there was some uncertainty around underestimation of levels of mortality and disease in Indigenous Australians due, in part, to under-enumeration of those of Indigenous identity and imprecise routine data collection systems [22]. OATSIH funded the IBoD study in an effort to improve the evidence base in Indigenous health and contribute to priority setting and resource allocation, and used the opportunity to source locally available global expertise in the GBD team.

There is widespread recognition of the value of policy being informed by evidence [25,26,41], and “*rigorous research findings are seen as useful and necessary inputs for policymakers in their ongoing consideration of policy development*” [26]. According to Head, three enabling factors underpin evidence-based policy: “*high-quality information based on relevant topic areas, cohorts of professionals with skills in data analysis and policy evaluation, and political incentives for utilising evidence-based analysis and advice in governmental decision-making processes*” [27]. All three of these enablers were arguably present and help explain why the study was commissioned.

The literature reminds us, however, that policymakers are not automatically attracted to drawing on research to inform their policy proposals: “*Clearly politics may affect how much notice policy makers take of research results. Where governments are committed to policy on ideological grounds, they may be only secondarily interested in research findings, especially if these challenge or question the policy impetus, its ideological basis or authoritative knowledge*” [28]. In the context of Indigenous health in Australia, Nutbeam and Boxall [29] argued that the Howard government (in power when the IBoD study was commissioned) placed its focus “*on addressing individual risk factors for ill health*”. This, too, would have been in keeping with the BoD approach and assessment of risk factors requiring intervention.

Research may be commissioned by governments to underpin a particular policy objective or direction, perhaps in support of an issue already on the policy agenda, or to shed light on a question for which the answer is unknown. It may also help to select between different intervention options. The Australian government at the time of the commissioning of the IBoD study was clearly committed to improving Indigenous health data and improving Indigenous health outcomes. Having research commissioned by government also increased the likelihood of utilisation of the products of that research by policymakers. As one policy advisor interviewed stated: “*… in terms of one of your questions about stakeholders and how to get policymakers involved, certainly as I was saying before, where the Department is involved in the development* [it] *is quite good. Like the development of* [the IBoD study] *– a lot of communication helps in terms of then implementing the findings*”. The literature bears this out, as stated by Davis and Howden-Chapman [42], who highlight the indirect links between research and policy, but state nevertheless that: “*researchers can reframe the way health policy issues are seen, and collaboration with policymakers initially can enhance implementation later*”.

Commissioning of the IBoD study was facilitated by the experiences of previous national and state-based BoD studies, as well as existing relationships between the government and researchers involved. Haynes et al. [43] note that existing relationships between policymakers and researchers are key to researchers influencing policy and “*good interpersonal relationships between researchers and policymakers are consistently identified as key facilitators of research-informed policy development*” (p. 1). An Australian study exploring the views and practices of policymakers and researchers with respect to the use of evidence in policy also found that existing relationships and networks with policymakers facilitated use of research evidence [44]. Relationships such as these clearly played a considerable role in the commissioning of the IBoD study, as did existing networks which are often the route through which policymakers engage researchers [43,45].

Across the range of informants interviewed, many iterated at least one of three related areas of anticipated benefit: informing policy, improving the evidence base, and priority setting. These findings demonstrate a number of shared interests amongst the range of stakeholders in having the IBoD study undertaken.

These expectations identified highlight an approach to policymaking that places emphasis on numbers and research data. It suggests a ‘rational’ approach to policymaking, seeing it as a somewhat technical activity in which numbers and quantification play a key part. The IBoD study, especially in concept, offered the potential to generate much desired ‘answers’ in the form of numbers. It seems that hard numbers, and an assumed relatively ‘objective’ method, were seen as helping advance an area in which few significant gains had been made, that was contentious politically, and for which new thinking was required. It was clearly hoped that the results of the study would feed into determining priorities and informing policy. The ranking of a range of conditions was, and still remains, a prominent feature of BoD studies globally and nationally, so if policy is seen as reflecting the allocation of effort and resources to achieve a particular outcome, the potential importance of such data in influencing Treasury and the allocation of funds to key areas of Indigenous health is apparent.

To some extent the commissioning of the IBoD study could be seen as addressing Kingdon’s three streams: problem, politics, and policy [30], coming together in the hope that the study would offer answers and help progress in the field. There was clear recognition of a problem – inequalities in health status of Indigenous Australians, a desire to find policy solutions that would make a difference, and bipartisan political support for addressing Indigenous disadvantage. The IBoD study offered new metrics and the potential to identify key areas for future investment and intervention.

Whilst many of those interviewed noted at least one of the three key expectations identified, several interviewees held slightly differing or completely opposing views. This may be indicative of their personal experiences with BoD studies, organisational base and role, prior views on what needed to be done to improve Indigenous health in Australia, or their views of the value of commissioned research and quantitative data to policy decision-making. Furthermore, as noted previously, critiques of the IBoD study by some informants were that existing data would not support the study, that numbers would not address the underlying issues of concern (which were dominated by socio-political concerns and social determinants of health), and that findings might compete with other available estimates.

A key limitation during the commissioning process, likely to influence the uptake and use of the IBoD study, was the noted lack of Indigenous engagement with the study. While some efforts were made to involve Indigenous academics and organisations within the commissioning and oversight of the study, those that were involved were there in a personal capacity and informal policy role. The one official Aboriginal community-controlled organisation invited to participate was unable to do so. As noted previously, this is likely to have influenced how the study may have been conceptualised and positioned within the policy debates around Indigenous health inequalities.

A comment on limitations within our study is also appropriate to note. These insights were derived primarily from a series of interviews undertaken several years after the IBoD study was published, and may have therefore been influenced by informant views on the study outcomes, in this way differing from their views at the time. While reflecting post-hoc analyses, rather than real-time, these were supplemented by the literature of the time and other relevant documentation. While many informants were willing to share potentially sensitive insights and data, other ‘behind-the-scenes’ views may not have been shared with us.

## Conclusions

The Indigenous health context in the lead-up to the commissioning of the IBoD study included concern around longstanding Indigenous disadvantage, shown through high mortality and morbidity differentials, lower life expectancy than non-Indigenous Australians, and the perceived lack of an adequate evidence base upon which to determine priorities for interventions. It was within this broad context that the IBoD study was commissioned. We identified several anticipated benefits and expectations of key stakeholders, including Indigenous and non-Indigenous academics and bureaucrats from a range of institutional bases. Many informants noted that they hoped the study would inform the evidence base, contribute to priority setting, and/or inform policy. The IBoD study offered the potential to generate much desired ‘answers’, in the form of numbers, and it was anticipated by many that the results of the study would feed into determining priorities and informing Indigenous health policy. A notable lack, however, was the full engagement of Indigenous organisations, which may have influenced how the study outcomes were subsequently used. A recently commissioned second IBoD study, led by the Australian government utilising 2013 data, may well approach this process differently.

## Endnotes

^a^Australian Institute of Health and Welfare; Department of Health and Ageing; Office for Aboriginal and Torres Strait Islander Health; National Aboriginal Community Controlled Health Organisation; National Advisory Group on Aboriginal and Torres Strait Islander Health Information and Data; Council of Australian Government’s; Australian Institute of Aboriginal and Torres Strait Islander Studies; The Department of the Prime Minister and Cabinet.

^b^One of the initiators of the GBD study and methodology, and member of the research team that undertook the Australian national and IBoD studies.

## Abbreviations

ABS: Australian Bureau of Statistics
AIHW: Australian Institute of Health and Welfare
BoD: Burden of disease
DALY: Disability-adjusted life years
DOHA: Australian Government Department of Health and Ageing
GBD: Global Burden of Disease
IBoD: Indigenous Burden of Disease
NACCHO: National Aboriginal Community Controlled Health Organisation
OATSIH: Office for Aboriginal and Torres Strait Islander Health
SC: Steering committee
TAP: Technical advisory panel
UQ: University of Queensland
